# Varied behavioral responses induced by morphine in the tree shrew: a possible model for human opiate addiction

**DOI:** 10.3389/fnbeh.2014.00333

**Published:** 2014-09-23

**Authors:** Fang Shen, Ying Duan, Shubo Jin, Nan Sui

**Affiliations:** ^1^Key Laboratory of Mental Health, Institute of Psychology, Chinese Academy of SciencesBeijing, China; ^2^Institute of Psychology, University of Chinese Academy of SciencesBeijing, China

**Keywords:** tree shrews, morphine, locomotor activities, conditioned place preference/aversion (CPP/CPA), self-administration

## Abstract

Tree shrews represent a suitable animal model to study the pathogenesis of human diseases as they are phylogenetically close to primates and have a well-developed central nervous system that possesses many homologies with primates. Therefore, in our study, we investigated whether tree shrews can be used to explore the addictive behaviors induced by morphine. Firstly, to investigate the psychoactive effect of morphine on tree shrews’ behavior, the number of jumping and shuttling, which represent the vertical and horizontal locomotor activity respectively, was examined following the injection of different dosage of morphine. Our results showed intramuscular (IM) injection of morphine (5 or 10 mg/kg) significantly increased the locomotor activity of tree shrews 30–60 min post-injection. Then, using the conditioned place preference/aversion (CPP/CPA) paradigm, we found morphine-conditioned tree shrews exhibited place preference in the morphine-paired chamber on the test day. In addition, naloxone-precipitated withdrawal induced place aversion in the chronic morphine-dependent tree shrews. We evaluated the craving for morphine drinking by assessing the break point that reflects the maximum effort animals will expend to get the drug. Our data showed the break point was significantly increased when compared to the baseline on the 1st, 7th and 14th day after the abstinence. Moreover, in the intravenous morphine self-administration experiment, tree shrews conditioned with morphine responded on the active lever significantly more frequently than on the inactive lever after training. These results suggest that tree shrew may be a potential candidate for study the addictive behaviors and the underling neurological mechanisms.

## Introduction

Preclinical studies involving animal behavior have been pivotal for providing insights into the psychobiological substrates of drug addiction, the understanding and knowledge of which increase in parallel with the refinement of animal models of this pathology. Rodents (mainly rats and mice) are popular animal models for drug addiction research because of their small size, rapid propagation, the availability of a variety of inbred strains, and transgenic techniques. However, there are some differences between rodent species and humans particularly in terms of genetics, pathology, and pharmacology. These differences make it difficult to translate data to the human paradigm and have limited application in further studies on this topic. Also, the addiction-like behaviors appear in a small proportion (about 17%) of rats using addictive drugs in the self-administration paradigm, indicating that this paradigm might need higher cognitive skills to complete the complex operation (Deroche-Gamonet et al., 2004). Therefore, more appropriate animal is required to investigate the addictive behavior induced by addictive drugs. A recent whole genome sequencing study confirms that the Chinese tree shrew has a closer affinity to nonhuman primates than rodents, indicating they may be more suitable for the study of the pathogenesis of human disease (Fan et al., 2013). Moreover, present studies indicate that the neurochemical characterization of the tree shrew striatum, especially dorsal striatum (Rice et al., 2011) and nucleus accumbens (McCollum and Roberts, 2014), more closely resembles that of the primate than the rodent.

We thus investigated whether tree shrews can be used to explore the addictive behavior induced by morphine. Since no previous studies have reported the psychoactivity to the stimulant effects of morphine on the tree shrews’ behavior, we first explored the locomotor activity after the injection of different doses of morphine. Then, we explored the possibility of establishing the addiction models in tree shrews using the conditioned place preference (CPP)/aversion (CPA) paradigm, which is widely used to study the rewarding or aversive effects of drugs. In this part of the experiment, we investigated whether morphine-conditioned tree shrew could spend more time in the morphine-paired chamber after training. Then, we also investigated whether the chronic morphine-dependent tree shrews could spend significantly less time in the naloxone-paired chamber on the test day.

Although some authors suggest that CPP is a model of drug seeking (or craving) behavior, CPP alone cannot account for the instrumental nature of drug seeking and drug taking behavior, which is perhaps better modeled by drug self-administration procedures. It is known that tree shrews have a high brain-to-body mass ratio and are able to use their forepaws to climb trees and handle food, indicating they may be more competent for complex operations such as self-administration (Steele, 1982). Therefore, in the next experiment, we established morphine self-administration models in tree shrews. Firstly, we used the break point to examine the craving of tree shrews for morphine drinking on the 1st, 7th and 14th day after abstinence vs. the baseline. In the progressive ratio (PR) schedule of self-administration, the point in the series at which responding ceases is labeled the break point and presumably reflects the maximum effort that animals will expend to get addictive drug. So, the increase of the break point corresponds to a stronger craving for the drug. Then, using the intravenous self-administration paradigm, we further evaluated the self-administrating behavior in tree shrews induced by morphine.

In short, the present study was designed to determine whether tree shrews can be used to explore the addictive behavior induced by morphine, and then provide a candidate species for studying the neurological mechanism of drug addiction in the future.

## Materials and methods

### Animals

This study used adult male tree shrews (*Tupaia belangeri chinensis*) weighing 120–170 g. These tree shrews were obtained from the breeding colony at the Animal House Center of the Kunming Institute of Zoology. Animals were individually housed in stainless cages (395 × 300 × 595 mm) attached to the nest boxes (246 × 158 × 147 mm). The nest boxes provided sleeping quarters and were also used as transfer boxes when the animals were moved from their home cages to the training apparatus. Under normal conditions, the round door of the nest box was open to allow the tree shrews to move about freely. During the experiments, the round door was closed to secure the animals in the nest box and transfer them. Tree shrews were kept in a temperature controled room and maintained on a standard 12-h light/dark cycle (light on at 08:00). Food and water were provided *ad libitum*. All behavioral tests were conducted during the light phase. All the procedures were conducted according to the National Institutes of Health Guide for the Care and Use of Laboratory Animals, and the protocols were approved by the Research Ethics committee of Institute of Psychology, Chinese Academy of Sciences.

### Drugs

Morphine hydrochloride (Qinghai Pharmaceutical, China) and naloxone hydro- chloride (Sigma, Missouri, USA) were dissolved in sterile physiological saline (0.9% NaCl) to its final concentrations. Sucrose was dissolved in distiled water for a stock concentration of 10%. Morphine was dissolved in 10% sucrose solution for a stock concentration of 2 mg/ml as morphine sucrose solution.

### Apparatus

#### Conditioned place preference/aversion

Tree shrews, unlike rodents, have a well-developed visual system and they can distinguish different colors such as blue, green, etcetera. Therefore, the conditioned training equipment we designed was mainly dependent on the color cues of the different sides of chambers. In our present experiments, the CPP/CPA apparatus was composed of two stainless steels chambers designated as chamber A and chamber B attached to the nest boxes. Chamber A had yellow and white horizontal stripes on the walls (4 cm wide) and a yellow floor. There was a yellow circular block (*d* = 40 mm, *D* = 80 mm) hanging on the wall next to the nest box, the floor of which was smooth. Chamber B had blue and white vertical stripes (4 cm wide) and a blue floor. There was a blue circular block (*d* = 40 mm, *D* = 80 mm) on the wall next to the nest box, the floor of which was coarse. Both chambers had cameras mounted on the top to record the animals’ behavior. Between the two chambers there was a middle wall that could be opened.

#### Oral morphine self-administration

The apparatus was a cage attached to the nest box and a camera mounted on the top. A fixed response lever (SED-121, Anilab) decorated with green paper and a feed box was mounted on the lateral wall of the cage. Drug solution was delivered by a 10 ml syringe in an infusion pump (SED-211, Anilab). Experimental sessions were controled and recorded using Anilab PC ver 6.50.

#### Morphine self-administration

The tree shrews were trained and tested for morphine self-administration in standard operant chambers (Med Associates, Inc., St. Albans, VT, USA), which were placed in a sound-attenuating cubicle. Each chamber contained two levers (ENV-114M, Med Associates), located 5 cm above the grid floor. Yellow or green paper was placed on the two levers respectively as a visual cue and pink or green feathers was pasted on the lever to attract the tree shrew’s interest. Drug solution was delivered through polyethylene tubing, protected by a leash assembly (PHM-120, Med Associates) and suspended through the ceiling of the chamber from a fluid swivel (PHM-115, Med Associates). Drug was delivered by a 10 ml syringe in an infusion pump (PHM-100, Med Associates). Experimental sessions were controled and recorded using MED PC Software IV (Med Associates).

### Procedure

#### Locomotor activity

To explore the effect of different doses of morphine on the tree shrews’ locomotor activity, we tested the animals in observation cage attached to the nest boxes. The observation cage is the same with the home cage in size and material. On day 0, all the tree shrews were allowed to habituate in the observation cage for 1 h. Then, the tree shrews were divided randomly into morphine and saline groups. They were intramuscularly (IM) injected with saline (2 ml/kg, IM) or escalating doses of morphine (5, 10 and 20 mg/kg for 3 days, each day with one dose and each dose was given three times a day). The interval of time between each injection was 4 h. A camera was mounted on the top of the observation cage and recorded the animals’ behaviors. The videos were analyzed to determine which behavior could represent animals’ activity post-injection of morphine. The number of vertical jumping (the vertical activity) including somersaulting and climbing and the number of shuttling between the cage and nest box (the horizontal activity) were counted by observers.

#### Conditioned place preference

##### Pre-test

The middle wall was opened (5 cm wide) and the tree shrews were placed in one lateral nest box 1 day and placed in the other lateral nest box the next day to explore the entire apparatus for a 1 h daily session from day −1 to day 0. When the tree shrews were placed into the apparatus, they might hide in the nest box and did not explore the area for a while. Therefore, data acquisition started when the tree shrews were first out of the nest box and lasted for 30 min. The time spent in each chamber on day −1 and 0 was recorded. The apparatus was considered to be unbiased to tree shrews’ preference. So, the data from pre-tests were used to separate animals into groups with equal biases for each chamber.

##### Conditioning

From day 1 to 10, the middle wall was closed. On the first day, the morphine group animals were confined to one lateral chamber (twice a day, 10:00 and 14:00) for 90 min after the injection of morphine (5 mg/kg, IM). On the second day, the tree shrews were injected with saline (2 ml/kg, IM) and confined to the other conditioned chamber. On the subsequent conditioning days, each tree shrew in morphine group was trained for 8 consecutive days with an alternate injection of morphine (10 mg/kg, IM) and saline (2 ml/kg, IM). To ensure a high psychoactive effect of morphine during training, the interval time between the morphine injection and placing the animal in the chamber was altered (30 min on day 1–2, 45 min on day 3–10). Animals in the control group received saline in both chambers every day. In the morphine group, half of the animals received morphine training in chamber A and saline training in chamber B, while the rest received morphine and saline in chamber B and A, respectively.

##### Test

On day 11 and 12, the middle of the wall was opened. The tree shrews were placed in the apparatus as the pre-test phase. The CPP score was defined as the time spent in the morphine-paired chamber divided by the total time spent in both chambers. During the CPP test, the number of vertical jumps and shuttles between the cage and nest box were estimated as the vertical and horizontal locomotor activity index, respectively. The number of shuttles between the two chambers was also recorded to examine the tree shrews’ exercise capacity.

#### Conditioned place aversion

##### Pre-test

The tree shrews were placed in one lateral nest box 1 day and placed in the other lateral nest box the next day to explore the entire apparatus for 1 h on day −1 and 0. The average time spent in each chamber was recorded for 30 min.

##### Chronic morphine treatment

From day 1 to day 6, the morphine group animals received escalating doses of morphine (5, 10, 15, 20, 25 and 25 mg/kg, IM, interval of 4 h twice a day). The control group animals received an equal volume of saline every day.

##### Conditioning

On day 7, the tree shrews were injected with morphine (25 mg/kg, IM) or saline at 10:00. 4 h later, all the tree shrews were injected with naloxone (1.25 mg/kg, IM) and placed in the drug-paired chamber for 90 min.

##### Test

On day 8 and 9, the middle wall was opened and the tree shrews were placed in the same manner as the pre-test. The time spend in each camber was recorded. The CPA score was defined as the time spent in the drug-paired chamber in the pre-test phase subtracted from the time spent in the drug-paired chamber in the test phase and then divided by the total test time (30 min).

#### Oral morphine self-administration

On day 1 and day 2 the tree shrews were tested for the break point of drinking morphine mixed with 10% sucrose solution or 10% sucrose solution at 11:00 and 15:00 (measuring sequence was balanced) under the PR69 procedure. The average break point of day 1 and 2 was the baseline data. In the following 10 days the tree shrews were administered escalating doses (5, 15, 20, 30, 45, 45, 60, 60, 75, 75 mg/kg) of morphine mixed with 10% sucrose in the bottles hanging on their own home cages. Then, tree shrews were tested for the break point of morphine mixed with 10% sucrose solution or 10% sucrose solution on the 1st, 7th and 14th day after the abstinence.

#### Intravenous morphine self-administration

##### Surgery

Tree shrews were anesthetized with sodium pentobarbital (100 mg/kg, i.p.; Sigma-Aldrich) before implantation with jugular catheters. A silicone catheter was inserted 28 mm into the right jugular vein and delicately anchored to the vein with silk suture. The other end of the catheter passed subcutaneously to exit into a 22-gauge connector (Plastics One, Roanoke, VA, USA) mounted on the back and was covered with a solid pin when not in use for drug infusions.

##### Intravenous morphine self-administration training

The tree shrews self-administered morphine in 12 continuous 3 h-sessions. Morphine was dissolved in saline and infused in a volume of 0.1 ml over 5 s at a dose of 0.5 mg/kg per infusion under a fixed-ratio 1 (FR1). Lever press in the active side resulted in an infusion of morphine and a presentation of a compound tone-light cue (5 s activation of the white stimulus light above the active lever side and a tone generator, 2 kHz, 70 dB, 10 dB above ambient noise) for 5 s, followed by a 20 s timeout period. The response during the timeout period was recorded but had no programmed consequences. Responses in the inactive lever side were counted but had no scheduled consequences during the full session.

### Statistical analysis

The data were analyzed with *t*-test, two-way ANOVA, and then followed by Bonferroni *post hoc* tests when appropriate. All data was shown as mean ± SEM and processed by the commercially available software Graph Pad Prism 5.0. The accepted level of statistical significance is *p* < 0.05.

## Results

### The effect of different doses of morphine on the tree shrews’ locomotor activity

In this experiment, the six tree shrews were randomly divided into morphine (*n* = 3) and saline (*n* = 3) group. The number of jumps and shuttles between the cage and nest box, which were considered as the vertical and horizontal locomotor activity indices, respectively, were recorded after the injection of different doses of morphine or saline.

**Figure 1:**
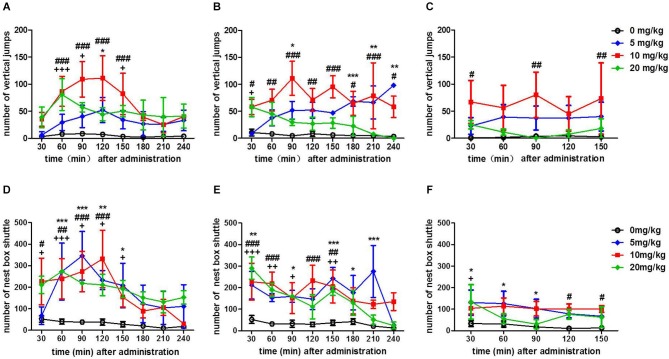
**Number of vertical jumps and horizontal shuttles between the cage and nest box after the injection of different doses (0, 5, 10, 20 mg/kg, IM) of morphine, which were considered as the vertical and horizontal activity indices, respectively**. The number of vertical jumps after the first time **(A)**, the second time (4 h after the first time) **(B)** and the third time (4 h after the second time) **(C)** administration of three doses of morphine. The number of horizontal shuttles between the cage and nest box after the first time **(D)**, the second time (4 h after the first time) **(E)** and the third time (4 h after the second time) **(F)** administration of three doses of morphine. “*”, “#” and “+” represent, respectively, the significant difference between 5, 10 or 20 mg/kg and 0 mg/kg morphine. *^/#/+^ *p* < 0.05, **^/##/++^ *p* < 0.01, and ***^/###/+++^ *p* < 0.001 vs. 0 mg/kg morphine. The data are expressed as means ± SEM, and analyzed using two-way ANOVA followed by Bonferroni *post hoc* test, *n* = 3.

After the first injection, the curve of the vertical activity of the tree shrews post-injection of the three doses of morphine showed an inverted U-shaped curve, whereas the saline-control group’s vertical activity did not change (Figure 1A). *Post hoc* analyses indicated that there was a significant increase in the number of vertical jumps induced by 5 mg/kg morphine in 90–120 min after the injection of morphine (*t* = 2.805, *p* < 0.05, vs. 0 mg/kg morphine); the number of vertical jumps induced by 10 mg/kg morphine increased significantly in 30–60, 60–90, 90–120 and 120–150 min after the injection (*t* = 4.895, *p* < 0.001; *t* = 6.234, *p* < 0.001; *t* = 6.443, *p* < 0.001; *t* = 4.882, *p* < 0.001, vs. 0 mg/kg morphine); the number of vertical jumps of 20 mg/kg morphine was also increase significantly in 30–60, 60–90 and 120–150 min after the injection (*t* = 4.523, *p* < 0.01; *t* = 3.031, *p* < 0.01; *t* = 2.918, *p* < 0.01, vs. 0 mg/kg morphine). Furthermore, the peak effect occurred during the second hour of 10 mg/kg morphine injection (Figure 1A). After the second injection, the curve of the vertical activity post-injection of 10 mg/kg still showed an inverted U-shaped curve, whereas the curve of 5 mg/kg and 20 mg/kg morphine injection showed a different trend 4 h post-injection. Post hoc analyses indicated that there was significant increase in the number of vertical jumps induced by 5 mg/kg morphine in 60–90, 150–180, 180–210 and 210–240 min after the second injection (*t* = 2.955, *p* < 0.05; *t* = 3.974, *p* < 0.001; *t* = 3.871, *p* < 0.01; *t* = 3.587, *p* < 0.01, vs. 0 mg/kg morphine); the number of vertical jumps induced by 10 mg/kg morphine significantly increased in 0–240 min after the injection (*t* = 2.976, *p* < 0.05; *t* = 3.932, *p* < 0.01; *t* = 6.683, *p* < 0.001; *t* = 3.936, *p* < 0.01, *t* = 5.608, *p* < 0.001; *t* = 3.744, *p* < 0.01; *t* = 4.646, *p* < 0.001; *t* = 3.183, *p* < 0.05, vs. 0 mg/kg morphine); in addition, there was also a significant increase in the number of vertical jumps in 0–30 min after the second injection of 20 mg/kg morphine (*t* = 2.955, *p* < 0.05, vs. 0 mg/kg morphine) (Figure 1B). After the third injection, except at 0–30, 60–90 and 120–150 min after the injection of 10 mg/kg morphine (*t* = 3.173, *p* < 0.05; *t* = 3.657, *p* < 0.01; *t* = 3.394, *p* < 0.01 vs. 0 mg/kg morphine), the vertical activity in all doses from the morphine group did not change vs. that in the saline control group (Figure 1C).

Next, we explored the effect of different doses of morphine on the horizontal locomotor activity. After the first injection, the curve of the horizontal activity post-injection of different doses of morphine showed an inverted U-shaped curve, whereas the saline control group’s horizontal activity did not change. Furthermore, the peak effect occurred during the second hour of 5 mg/kg morphine injection. *Post hoc* analyses indicated that there was significant increase in the number of shuttles between the cage and nest box in the 5 mg/kg morphine group at 30–60, 60–90, 90–120 and 120–150 min after the injection (*t* = 4.164, *p* < 0.001; *t* = 5.517, *p* < 0.001; *t* = 3.502, *p* < 0.01; *t* = 3.221, *p* < 0.05, vs. 0 mg/kg morphine); the number of shuttles between the cage and nest box of the 10 mg/kg morphine group increased significantly at 0–30, 30–60, 60–90 and 90–120 min after the injection (*t* = 3.122, *p* < 0.05; *t* = 3.567, *p* < 0.01; *t* = 4.203, *p* < 0.001; *t* = 5.257, *p* < 0.001, vs. 0 mg/kg morphine); In addition, there was also a significant increase in the number of shuttles between the cage and nest box at 0–30, 30–60, 60–90, 90–120 and 120–150 min after the injection of 20 mg/kg morphine (*t* = 2.836, *p* < 0.001; *t* = 4.188, *p* < 0.001; *t* = 3.224, *p* < 0.05; *t* = 3.096, *p* < 0.05; *t* = 2.934, *p* < 0.05, vs. 0 mg/kg morphine) (Figure 1D). After the second injection, the horizontal activity post-injection of all doses of morphine increased; whereas the saline control group’s horizontal activity did not change. *Post hoc* analyses showed that there was a significant increase in the number of shuttles between the cage and nest box in the 5 mg/kg morphine group at 0–30, 60–90 120–150, 150–180 and 180–210 min after the injection (*t* = 3.602, *p* < 0.01; *t* = 2.866, *p* < 0.05; *t* = 4.670, *p* < 0.001; *t* = 3.040, *p* < 0.05; *t* = 5.743, *p* < 0.001, vs. 0 mg/kg morphine); the number of shuttles between the cage and nest box of the 10 mg/kg morphine group increased significantly at 0–30, 30–60, 90–120 and 120–150 min after injection (*t* = 3.963, *p* < 0.001; *t* = 4.232, *p* < 0.001; *t* = 4.570, *p* < 0.001; *t* = 3.811, *p* < 0.01, vs. 0 mg/kg morphine). In addition, there was also a significant increase in the number of shuttles between the cage and nest box at 0–30, 30–60, 60–90 and 120–150 min after the injection of 20 mg/kg morphine (*t* = 5.395, *p* < 0.001; *t* = 3.569, *p* < 0.01; *t* = 2.851, *p* < 0.05; *t* = 3.359, *p* < 0.01 vs. 0 mg/kg morphine) (Figure 1E). After the third injection, *Post hoc* analyses showed that there was a significant increase in the number of shuttles between the cage and nest box in the 5 mg/kg morphine group of tree shrews at 0–30, 30–60 and 60–90 min after the injection (*t* = 3.040, *p* < 0.05; *t* = 2.957, *p* < 0.05; *t* = 2.648, *p* < 0.05, vs. 0 mg/kg morphine). The number of shuttles between the cage and nest box of the 10 mg/kg morphine group significantly increased at 90–120 and 120–150 min after the injection (*t* = 2.807, *p* < 0.05; *t* = 2.734, *p* < 0.05, vs. 0 mg/kg morphine). In addition, there was also a significant increase in the number of nest box visits at 0–30 min after the injection of 20 mg/kg morphine (*t* = 3.144, *p* < 0.05, vs. 0 mg/kg morphine) (Figure 1F).

The data indicated that the morphine increased the vertical and horizontal locomotor activity to a different degree, and the optimal morphine dose might be 10 mg/kg and the starting time of training was about 30–60 min after the first two IM injection of morphine (Figure 1).

### Establishment of morphine CPP

**Figure 2:**
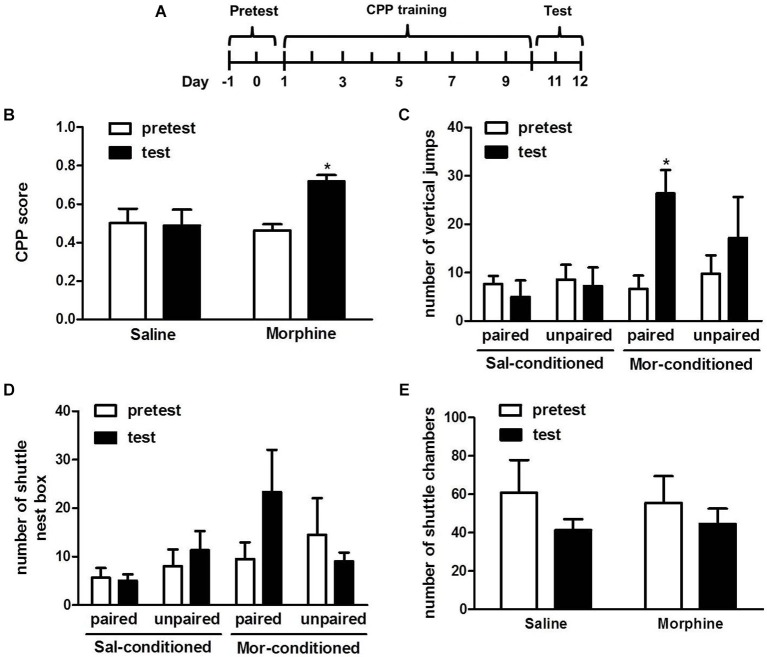
**Effects of morphine on the induction of conditioned place preference**. **(A)** Diagram outlining the behavioral procedures. **(B)** Tree shrews that received alternating injections of morphine and saline showed a significant preference for the morphine-paired chamber. **(C)** The vertical jump number of the morphine-conditioned tree shrews in the morphine-paired chamber significantly increased on the test day. **(D)** The amount of shuttles between the cage and nest box of morphine-conditioned tree shrews in the morphine-paired chamber had an increasing trend on the test day. **(E)** The amount of shuttles between the two chambers of morphine- and saline- conditioned tree shrews had no change. Blank and solid columns represent data from pre- and post-conditioning tests, respectively. * *p* < 0.05, pretest vs. test. The data are expressed as means ± SEM, and analyzed using two-way ANOVA followed by Bonferroni *post hoc* test, *n* = 5–6.

The 11 tree shrews were divided into morphine (*n* = 6) and saline (*n* = 5) groups randomly. 5 mg/kg dose of morphine was chosen as the first dose of injection to avoid too much stimulation and 10 mg/kg doses were given in the rest of the training sessions. After 10 days of alternative morphine and saline treatments or saline conditioning alone, the tree shrews underwent the CPP expression test on day 11 and 12 (Figure 2A).

Two-way ANOVA showed significant effects from the interaction between the treatments (morphine vs. saline) and the tests (pretest vs. test) (*F*_(1, 20)_ = 4.934, *p* < 0.05). But the pre-test vs. test and the difference between morphine- and saline-treated tree shrews were not significant. The followed Bonferroni *post hoc* test demonstrated that morphine-conditioned tree shrews spent significantly more time in the morphine-paired chamber (*t* = 2.985, *p* < 0.05, pre-test vs. test) (Figure 2B).

The vertical and horizontal activity of the tree shrews during the CPP test were also analyzed, the number of vertical jumps in morphine-conditioned tree shrews was significantly increased in the morphine-paired chamber (*t* = 3.160, *p* < 0.05, pre-test vs. test) (Figure 2C). In addition, the number of shuttles between the cage and nest box for the morphine-conditioned tree shrews had an increasing trend in the morphine-paired chamber on the test day than that on the pre-test day (Figure 2D). Moreover, the number of shuttles between chamber A and B was not changed on the test day, indicating the exercise capacity of tree shrews was basically stable (Figure 2E).

The data in Figure 2 showed that the morphine group spent significantly more time in the morphine-paired chamber compared with the saline-paired chamber after conditioned training, indicating our designed conditioning procedure successfully induced morphine CPP in tree shrews.

### Establishment of CPA in morphine-dependent tree shrews

**Figure 3:**
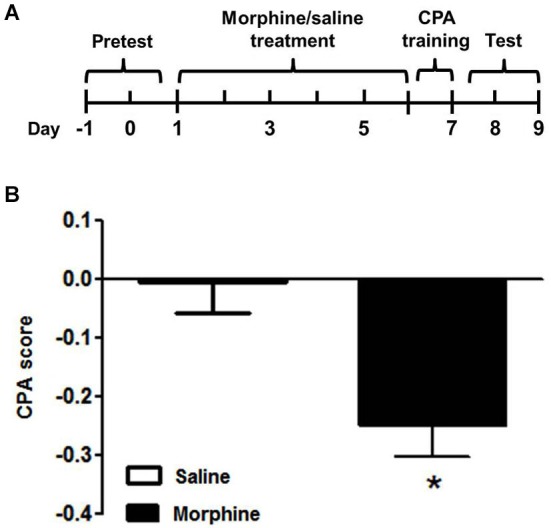
**Naloxone-induced conditioned place aversion in morphine-dependent tree shrews**. **(A)** Diagram outlining the behavioral procedures. **(B)** The morphine-dependent tree shrews exhibit place aversion to an environment previously paired with the withdrawal induced by naloxone, whereas the saline control group spent almost equal time in the two chambers. Blank and solid columns represent data from saline- and morphine-dependent tree shrews, respectively. The data are expressed as means ± SEM, *n* = 4–5. * *p* < 0.05, vs. the saline control group, *t*-test.

The nine tree shrews were divided into morphine (*n* = 5) and saline (*n* = 4) groups randomly. The morphine group received the escalating doses of morphine injection for 6 continuous days. The saline group was injected with an equal volume of saline. On day 7, all tree shrews received the injection of naloxone and were confined to the naloxone-paired chamber for 90 min. Then, the tree shrews underwent the CPA test on day 8 and 9 (Figure 3A). Statistical analysis (*t*-test) revealed that there were significant differences within the morphine- and saline- treated tree shrews (*t* = 3.205, *p* < 0.05) (Figure 3B).

The data in Figure 3 showed that compared with the saline group, the chronic morphine-dependent tree shrews spent significantly less time in the naloxone-paired chamber after training, indicating that naloxone successfully induced CPA in the morphine-dependent group.

### Establishment of oral morphine self-administration

**Figure 4:**
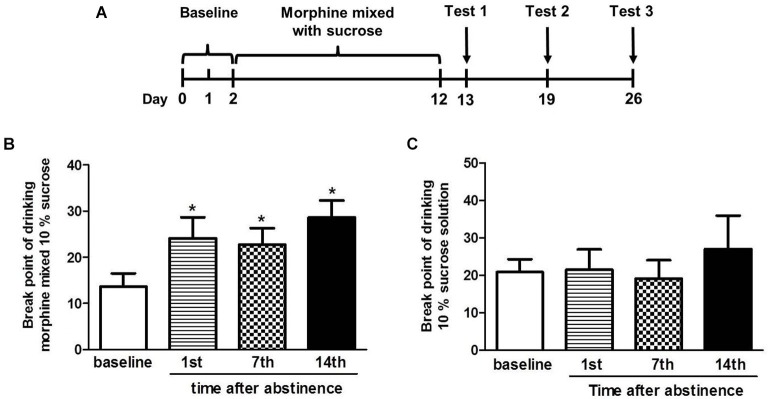
**The break point of drinking morphine mixed with 10% sucrose solution or 10% sucrose solution after the abstinence**. **(A)** Diagram outlining the behavioral procedures. **(B)** The break point of drinking morphine mixed with sucrose in the tree shrews was significantly increased when compared to the baseline on the 1st, 7th and 14th day after the abstinence. **(C)** The break point of drinking 10% sucrose in the tree shrews was not changed vs. the baseline at any of the three time point. The data are expressed as means ± SEM, *n* = 4. * *p* < 0.05, compare to the baseline, *t*-test.

In this experiment, we established the oral morphine self-administration model and explored the craving for the morphine after the abstinence in four tree shrews. On day 1 and 2, the break point of drinking morphine mixed with 10% sucrose or 10% sucrose solution alone was tested in tree shrews. After 10 continuous days of drinking the escalating doses of morphine mixed with a sucrose solution, the four tree shrews were tested for the break point of drinking morphine mixed with 10% sucrose or 10% sucrose solution on the 1st, 7th and 14th day after the abstinence (Figure 4A).

*t*-test analysis showed that significant differences in the break point of drinking morphine mixed with 10% sucrose were found between the baseline and the 1st day (*t* = 4.096, *p* < 0.05), the 7th day (*t* = 2.656, *p* < 0.05) and the 14th day (*t* = 3.397, *p* < 0.05) after abstinence (Figure 4B). However, there were no significant differences in the break point of drinking 10% sucrose between the baseline and the 1st day (*t* = 0.180, *p* > 0.05), the 7th day (*t* = 0.391, *p* > 0.05) and the 14th day (*t* = 0.648, *p* > 0.05) (Figure 4C) after the abstinence.

These data in Figure 4 showed that after the abstinence the break point of drinking morphine in tree shrews was significantly increased, whereas the break point of drinking sucrose was almost unchanged contrast to the baseline, indicating the tree shrews’ craving for drinking morphine was stronger after the abstinence.

### Establishment of intravenous morphine self-administration

**Figure 5:**
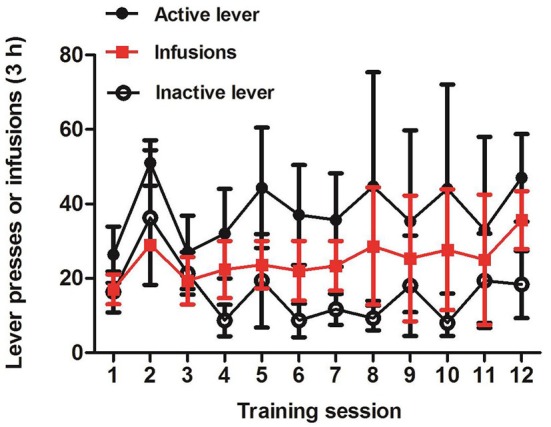
**Self-administrated behavior induced by intravenous morphine injection in tree shrews**. Mean ± SEM of infusions and response on the active or inactive lever during the 12 session days of morphine self-administration training. *p* = 0.0525, the active vs. the inactive responses, *t*-test, *n* = 3.

Figure 5 showed the mean ± SEM number of morphine infusions and response on the active or inactive levers for all the three tree shrews. After 12 consecutive days of training, *t*-test analysis demonstrated that there was an increase in active lever compressions compared to the inactive press (*p* = 0.0525). These findings displayed that, in the self-administration paradigm, tree shrews established the association between the active lever press and the intravenous injection of morphine, indicating that conditioned training induced self-administration behavior.

## Discussion

To our knowledge, our study was the first to demonstrate that tree shrews can be used for studying the effects of addictive drugs, such as morphine, on behavior using the CPP/CPA and self-administration paradigms.

**Figure 6:**
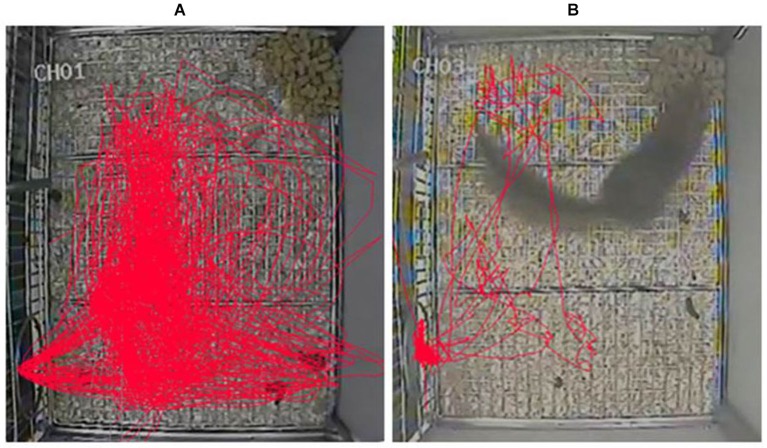
**Representative tracks of movements in tree shrews 30–60 min after the injection of 10 mg/kg morphine or saline**. **(A)** The tracing of a tree shrew’s movements 30–60 min after the injection of 10 mg/kg morphine. **(B)** The tracing of another tree shrew’s movements 30–60 min after the injection of saline (2 ml/kg).

Our present data showed that the first two injections of three doses of morphine resulted in significant increases in the vertical and horizontal activity 30–120 min post-injection, and the peak effect occurred during the second hour of 10 mg/kg morphine injection. These findings indicated that the optimal morphine dose might be 10 mg/kg and the starting time of training was about 30–60 min after the injection of morphine. In our pre-experimental period, we found there was an increase in the number of jumps and shuttles between the cage and nest box after the injection of morphine. And, we thought these two indexes might be used to measure the changes of locomotor activity in tree shrews. Just like the previous study used the tracing of tree shrews’ movements to examine novelty preference for the novel object (Khani and Rainer, 2012), we also found the moving distance was significantly increased after morphine injection (Figure 6). However, this measure might not fully reflect the changes of tree shrews’ activity in the vertical direction. Furthermore, our findings were partially in agreement with the results in rats that the lower dose of (1.25, 2.5 or 5 mg/kg) morphine significantly increased the vertical activity counts and had only an excitatory effect, whereas the higher doses (10 or 20 mg/kg) caused initial depression followed by a delayed excitatory effect (Babbini and Davis, 1972). It is worth noting that the three doses of morphine used in our study had only the excitatory effect in tree shrews, this result might be because the doses we used were not high enough.

Similar with previous work on the CPP paradigm in rats, the tree shrews exhibited place preference induced by morphine. What cannot be ignored is that the vertical jumping number of the morphine group animals was significantly increased in the morphine-paired chamber on the test day. Moreover, the number of shuttles between the cage and nest box also increased in the morphine-paired chamber. However, the number of shuttles between the two chambers was not changed, indicating the exercise capacity of tree shrews was basically stable. Our present findings suggested that tree shrews might be able to remember the special environment associated with morphine injection in which they experienced the excitatory effect induced by morphine previously, which resulted in their increased time spent and activities in the morphine-paired chamber. Furthermore, our data showed that the chronic morphine-dependent tree shrews produced CPA induced by naloxone, similar to previous findings in both tree shrews and rodents (Myers and Carlezon, 2010; Sun et al., 2012).

It is reported that short-term synaptic depression of the geniculo-cortical visual pathway was sensitized to morphine following chronic morphine exposure (Wang et al., 2006). The influence of morphine on this projection may contribute to the mediation of sensitized responses to drugs of abuse or environmental cues that trigger compulsive opiate seeking behavior leading to relapse (Wang et al., 2006). The above findings indicated that except for the ventral tegmental area and the striatum which are generally thought to be the major components of the reward system (Punch et al., 1997; Robbins and Everitt, 1999), the cortical visual pathway is also involved in the development of drug addiction. Consequently, a well-developed visual system (Jacobs and Neitz, 1986; Ranc et al., 2012) might place tree shrew as a valuable candidate to study the potential mechanisms responsible for the important role of the visual system in drug addiction.

Most previous behavioral studies show tree shrews have competent cognitive abilities. However, our present results in the CPP/CPA paradigm displayed tree shrews needed 10 training days, which is similar to rodents (Shen et al., 2012; Xu et al., 2012). We speculated that the competent cognitive abilities of tree shrews might be reflected in a longer retention time of addiction memory, just like the non-human primates, which can maintain this preference for at least 15.3 ± 1.7 months (Wang et al., 2012). In addition, the longevity of the tree shrew (about 5–7 years) is an important consideration, allowing for long-term studies to be conducted. Therefore, further studies will be required to explore the retention of reward memory using CPP paradigm in tree shrews.

Another common model for assessing the rewarding properties of addictive drugs is the self-administration paradigm. In the oral morphine self-administration experiment, we found the break point for morphine drinking was significantly increased compared to the baseline after the abstinence. This finding indicated that the initial aversions for the bitter morphine mixed with sucrose solution were converted into preferences after the tree shrews were repeatedly given the morphine mixed with sucrose solution to drink. It appeared that the post-ingestion effects of morphine provided primary reinforcement for the tree shrews and resulted in the consumption of more solution. However, the present data confused us because we did not know whether the increased consumption was due to the preference effect induced by morphine or tree shrews’ habituation to the bitter morphine solution mixed with sucrose. So, further control experiments must be produced to investigate the consumption of solution of quinine, which is equally bitter (aversive) to morphine. If the consumption of the quinine solution did not increase after repeated administration, we might be able to draw the conclusion that the increased break point was the result of the preference effect of morphine. Another question with the oral morphine self-administration paradigm was the bioavailability of morphine. It is reported that the oral bioavailability of morphine is just about 30% (Westerling et al., 1995). Though the oral administration is one of the most frequently used routes of drug administration, the oral route is problematic because of the unpredictable nature of gastro-intestinal drug absorption.

We thus further explored the addictive behavior in tree shrews using the intravenous self-administration paradigm. After 12 continuous sessions training, the tree shrews produced the preference for the active device, against the inactive one, attesting to the fact that morphine was self-administered. This self-administrating behavior was consistent with previous studies in rodents and non-human primates (Morgan et al., 2002; Porrino et al., 2004; Wee et al., 2007). However, the tree shrews’ response on the active lever was not very stable (the standard error was relatively big), and the number of compressions of the inactive lever did not drop to a very low level. In response to this phenomenon, we thought the reason might be the lever pressing itself was not a very appropriate operation mode for tree shrews because we found when they were jumping in the training chambers they would press the lever (active or inactive) inadvertently using some part of their body (such as their tails). Another possible reason might be that the apparatus was small for tree shrews, making it likely for them to press the levers unintentionally. This inadvertent and non-initiative lever pressing made tree shrews cannot differentiate between the lever pressing operation and the result of morphine injection. We also employed another operation mode, nose poke, to train tree shrews in the cocaine self-administration paradigm. According to our observation, there was almost no response on the nose poke inadvertently (data not shown). But due to the different addictive drugs (morphine vs. cocaine) in the current experiments we could not conclude certainly which mode is better. Therefore, more experiments are needed to develop the appropriate operation mode for tree shrews in the self-administration paradigm.

In summary, using the CPP/CPA and self-administration paradigms, we demonstrated that the tree shrew is a potential candidate for studies of both the addictive drugs effects on behavior and the neurochemical mechanisms underlying drug abuse in the future.

## Author contributions

Fang Shen and Ying Duan designed the experiments, preformed the experiments, analyzed data and wrote the paper; Shubo Jin preformed the experiments and analyzed data; Nan Sui designed the experiments and had primary responsibility for final content.

## Conflict of interest statement

The authors declare that the research was conducted in the absence of any commercial or financial relationships that could be construed as a potential conflict of interest.
